# Venlafaxine vs. fluoxetine in postmenopausal women with major depressive disorder: an 8-week, randomized, single-blind, active-controlled study

**DOI:** 10.1186/s12888-021-03253-8

**Published:** 2021-05-19

**Authors:** Jingjing Zhou, Xiao Wang, Lei Feng, Le Xiao, Rui Yang, Xuequan Zhu, Hui Shi, Yongdong Hu, Runsen Chen, Philip Boyce, Gang Wang

**Affiliations:** 1 The National Clinical Research Center for Mental Disorders & Beijing Key Laboratory of Mental Disorders & Beijing Anding Hospital, Capital Medical University, No 5. Ankang Lane, Deshengmen Wai, Xicheng District, Beijing, 100088 China; 2 Advanced Innovation Center for Human Brain Protection, Capital Medical University, Beijing, China; 3 Department of Clinical Psychology, Beijing Chao-Yang Hospital, Capital Medical University, Beijing, China; 4 Department of Psychiatry, University of Oxford, Oxford, UK; 5 Discipline of Psychiatry, Westmead Clinical School, Sydney Medical School, The University of Sydney, Sydney, NSW Australia; 6 Department of Psychiatry, Westmead Hospital, Sydney, Australia

**Keywords:** Fluoxetine, Postmenopausal depression, Randomized controlled trial, Venlafaxine

## Abstract

**Background:**

In the population of postmenopausal patients with major depressive disorder (MDD), the superiority of serotonin-norepinephrine reuptake inhibitors (SNRIs) over selective serotonin reuptake inhibitors (SSRIs) has not yet been definitively proven. Consequently, a direct comparison of the efficacy of SSRIs and SNRIs in the treatment of postmenopausal depression could provide relevant data. The aim of this study was to compare the efficacy and safety of venlafaxine vs. fluoxetine in the treatment of postmenopausal MDD.

**Methods:**

This was an 8-week, multicenter, randomized, single-blind, active-controlled trial conducted at a psychiatric hospital (Beijing Anding Hospital) and a general hospital (Beijing Chaoyang Hospital) between April 2013 and September 2017. The primary outcome measure was improving depressive symptoms (Hamilton Depression Rating Scale (HAMD-24) score). The secondary outcomes included the change of HAMD-24 anxiety/somatization factor score and Clinical Global Impressions-Improvement (CGI-I) response rate. Safety was assessed by treatment-emergent adverse events (TEAEs) and laboratory tests. Efficacy was analyzed by using the full analysis set (FAS) following the modified intention-to-treat (mITT) principle. The primary endpoint measurements were analyzed using a mixed-effect model for repeated measures (MMRM) model with patients as a random-effect factor, treatment group as the independent variable, time as a repeated measure, and baseline covariates, using a first-order ante dependence covariance matrix.

**Results:**

A total of 184 women were randomized. The full analysis set (FAS) included 172 patients (venlafaxine, *n* = 82; fluoxetine, *n* = 90). Over the 8-week study period, the reduction in HAMD-24 scores was significant (*P* < 0.001) in both groups, while a significantly greater decline from baseline was observed in the venlafaxine group compared with the fluoxetine group (least-squares mean difference [95% CI]: − 2.22 [− 7.08, − 0.41]), *P* = 0.001). The baseline-to-week-8 least-squares mean change of the anxiety/somatization factor scores, CGI-I response rate were greater in the venlafaxine group than in the fluoxetine group (all *P* < 0.05). The most frequent TEAEs (≥5%) in both groups were nausea, somnolence, dizziness, headache, and dry mouth. There was no significant difference in the incidence of adverse events between the two groups.

**Conclusion:**

Venlafaxine was well tolerated and compared to fluoxetine, it led to a greater improvement in the treatment of postmenopausal MDD.

**Trial registration:**

Clinical Trials. gov #NCT01824433. The trial was registered on April 4, 2013.

## Background

Major depressive disorder (MDD) is a common type of depressive disorder characterized by a persistent low mood, a lack of positive affect, and a loss of interest in usually pleasurable activities (anhedonia) that is different from the patient’s usual self and causes significant distress or impairment for ≥2 weeks [1, 2]. The prevalence of MDD in the United States is approximately 7% per year, with a lifetime prevalence of 16.6% [3]. It is estimated that 4.4% of the global population suffers from depression, which is the leading cause of disability worldwide [4].

It is well-known that females are more prone to depression than males. In fact, it is generally believed that compared to men, twice as many women experience major depression. Recent estimates of the global 12-month prevalence of major depressive disorder are 5.8% in women and 3.5% in men [5]. Postmenopausal women are at significant risk for depression [6]. A previous study has shown that 5.7% of women are diagnosed with MDD after menopause [7]. Treatment usually includes pharmacological therapy with antidepressants, such as selective serotonin reuptake inhibitors (SSRIs) and serotonin-norepinephrine reuptake inhibitors (SNRIs). Research suggests that postmenopausal and older women seem not to respond well to SSRIs as compared to pre- or perimenopausal women [8, 9]. On the other hand, SNRIs’ efficacy is consistent across age groups [10, 11]. These studies’ limitations are that the menopausal status was determined by age, a small sample size, and non-randomized designs. In the population of postmenopausal patients with MDD, SNRIs’ superiority over SSRIs has not yet been finally confirmed.

Venlafaxine is the first of the SNRIs that provides dose-dependent norepinephrine reuptake inhibition; a dosage of 150 mg/day or higher is sufficient to produce noradrenergic activity, and it has low affinity for the postsynaptic receptors [12, 13]. Fluoxetine is an SSRIs widely used for depressive disorders [14] that does not appreciably inhibit norepinephrine and dopamine reuptake at therapeutic doses. As it is the most commonly studied of the SSRIs, we elected to use it to compare it against venlafaxine.

Therefore, this randomized, single-blind trial compared the efficacy of venlafaxine and fluoxetine in postmenopausal women with MDD, aiming to evaluate the effect of postmenopausal status on the effectiveness of antidepressants. We hypothesized that venlafaxine has superior efficacy compared to fluoxetine for postmenopausal depression after 8 weeks.

## Methods

### Study design

This was an 8-week, multicenter, randomized, single-blind, active-controlled trial conducted at a psychiatric hospital (Beijing Anding Hospital) and a general hospital (Beijing Chaoyang Hospital) between April 2013 and September 2017. The study protocol was approved by the ethical review board at each study center. The study was carried out according to the Declaration of Helsinki and the guidelines for good clinical practice. The trial was registered (Clinical Trials. gov #NCT01824433), prior to the study. Written informed consent was obtained from all participants before the commencement of any study procedures. Patients were informed that they were free to withdraw from the trial at any time.

### Participants

Outpatient participants had to be ≥50 years old and to meet the Diagnostic and Statistical Manual of Mental Disorders-IV (DSM-IV) criteria for MDD, as determined by the Mini International Neuropsychiatric Interview (MINI). Menopausal status and menopausal age were self-reported. Menopause was defined as at least 1 year without menses. MDD could be newly-diagnosed with depressive episodes within 1 year or recurrent with at least 5 years from the last depressive episodes. Patients were required to have a 24-item Hamilton Depression Rating Scale (HAMD-24) total score ≥ 20, and HAMD item 1 (depressed mood) score ≥ 2 at screening and baseline. The exclusion criteria were: 1) current diagnosis of DSM-IV-TR axis I psychiatric illness such as schizophrenia, bipolar disorder, mental retardation, brain organic mental disorders, mental disorders due to a general medical condition, and any substance abuse disorder; 2) clinically significant medical diseases, including any cardiovascular, hepatic, renal, respiratory, hematologic, endocrinological, and neurologic diseases; 3) clozapine use up to 3 months before screening; 4) history of lacked efficacy of venlafaxine or fluoxetine; 5) not receiving hormonal replacement therapy; 6) treatment with electroconvulsive therapy (ECT) within 3 months prior to screening.

### Randomization and blinding

Patients meeting the eligibility criteria at baseline were randomly allocated to fluoxetine or venlafaxine at a 1:1 ratio using a computer-generated list of random numbers. In order to ensure allocation concealment, sequentially numbered opaque sealed envelopes were used. Individuals were randomized to groups by a researcher not connected to the study team. The raters who performed the evaluations were blinded to individual treatment assignments. All other researchers and the patients were unblinded to treatment group assignment.

### Treatment

The doses of venlafaxine and fluoxetine were tailored based on clinical considerations determined by the investigators at each site. The patients randomized to oral venlafaxine were initiated at a dose of 75 mg/d at the investigators’ considerations. Based on response and tolerability, the dose could be titrated upward to 150 mg/d at a 2-week interval, while the maximal dose was 225 mg/d. The patients randomized to oral fluoxetine were initiated at an initial dose of 20 mg/d, and the dose could be titrated upward by 10-mg increments at 2-week intervals according to investigators’ judgment to a maximum daily dose of 60 mg. The total study period was 8 weeks. The patients were examined at baseline and at weeks 1, 2, 4, 6, and 8.

The participants were not allowed to take any treatment that may affect the study drug’s efficacy, such as other psychotropic drugs, ECT, and psychotherapy. Benzodiazepines (preferred lorazepam) were prohibited 8 h before the psychiatric evaluations. Trihexyphenidyl could be used for extrapyramidal symptoms but not for prophylaxis, to a maximal dose of 12 mg/d during the study.

### Endpoints

The primary efficacy endpoint was the mean change in the HAMD-24 scores from baseline to week 8. The secondary efficacy endpoints were: 1) the mean change in the anxiety/somatization factor scores (sum of items 10–13, 15, and 17); 2) the proportion of Clinical Global Impressions-Improvement (CGI-I) score of 1 or 2 (very much or much improved) at week 8.

The HAMD-24 is a questionnaire used to provide an indication of depression in adults and to evaluate recovery and remission [15, 16]. The CGI-I scale is a investigators’ assessment of how much the illness improved or worsened in relation to baseline [17].

All patients underwent a complete physical examination at the beginning of the trial and at each subsequent visit. Safety was assessed based on the adverse events (AEs) recorded by the investigators. Electrocardiogram (ECG) recordings were performed at baseline and at 8 weeks. Blood pressure and heart rate were assessed at each study visit.

### Statistical analysis

The sample-size calculation showed that using a power of 90%, a total of 160 patients (80 in each treatment group) were required to demonstrate the superiority of venlafaxine to fluoxetine, with α set at 5%. Assuming a drop-out rate of 20%, enrolling 200 patients (100 in each group) was necessary. This was based on a superiority comparison of the treatment groups in HAMD-24 total score using a two-sided 95% confidence interval against a margin of 1.8 points, a standard deviation of 3.5 [18], and an expected true mean difference of 0 points between treatments.

Efficacy was analyzed using the full analysis set (FAS) following the modified intention-to-treat (mITT) principle, which included all randomized women who met the study criteria and took at least one dose of study drug and who had one or more post-baseline HAMD-24 evaluations. Per-protocol (PP) analysis refers to inclusion in the analysis of only those patients who strictly adhered to the protocol. Safety analyses were based on the safety set (SS), comprising all randomized patients who took at least one dose of study medication.

The endpoint measurements were analyzed using a mixed-effect model for repeated measures (MMRM) with patients as a random-effect factor, treatment group, time and research center as the fixed variable, patients as the random variable, random effects include intercept, baseline as a covariable, research center*group, time*group as interaction effect using a first-order ante dependence covariance matrix. Missing values were not imputed. The cumulative CGI-I response rate (much or very much improved) were compared between the two groups using the Fisher exact test. Categorical variables were described using frequencies (percentages) and continuous variables as means ± standard deviation (SD). Data were analyzed with SPSS software version 22 (IBM, Armonk, NY, USA). All statistical tests were two-sided. *P* values < 0.05 were considered statistically significant.

## Results

### Patients

**Fig. 1:**
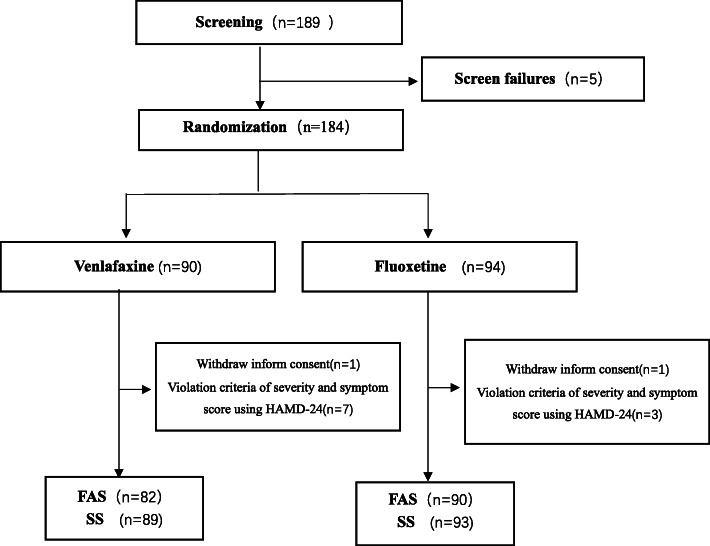
Patient flowchart. ^a^.HAMD-24, 24-item Hamilton Depression Rating Scale; FAS, full analysis set; SS, safety analyses

Figure 1 presents the patient flowchart. A total of 189 women were screened: five participants failed screening, and 184 women were randomized. The clinical and socio-demographic characteristics of the participants are presented in Table 1. There were no statistically significant differences between the two groups. Among them, 130 (63 on venlafaxine and 67 on fluoxetine) completed the 8-week study period; 26/90 (28.9%) participants in the venlafaxine group and 28/94 (29.8%) in the fluoxetine group dropped out from the study (*P* = 0.894). A total of 54 patients were discontinued from the study. The main reasons for discontinuation were participant decision (*n* = 11), poor adherence (*n* = 14), intolerable side effects (n = 11), loss to follow-up (*n* = 9), lack of efficacy (*n* = 5) and other reasons (*n* = 4). The mean daily doses for venlafaxine and fluoxetine at week 8 were 141.96 (55.3) mg/day and 38.96 (12.59) mg/day, respectively.

**Table 1:** Baseline characteristics of postmenopausal patients with major depressive disorder

Characteristics	Venlafaxine (*n* = 82)	Fluoxetine (*n* = 90)	*P*
Age (years), mean ± SD	56.7 ± 6.8	56.9 ± 5.8	0.83
BMI (kg/m^2^), mean ± SD	23.9 ± 3.8	23.0 ± 2.9	0.14
Age at onset (years), mean ± SD	52.3 ± 7.9	51.5 ± 8.2	0.55
Duration of illness (years), mean ± SD	5.7 ± 6.2	6.3 ± 6.9	0.54
Previous episodes, mean ± SD	2.0 ± 1.9	2.0 ± 2.3	0.88
HAMD-24 total score, mean ± SD	30.5 ± 7.3	31.7 ± 8.5	0.34
Anxiety/somatization score, mean ± SD	8.3 ± 2.6	8.8 ± 2.5	0.22
CGI-I score, mean ± SD	3.7 ± 1.2	3.7 ± 1.0	0.78
Family history, n (%)	10 (12.2)	9 (10.0)	0.65

*BMI* body mass index, *CGI-I* Clinical Global Impression of Improvement, *HAMD-24* 24-item Hamilton Depression Rating Scale, *SD* standard deviation

### Efficacy

#### FAS

**Fig. 2:**
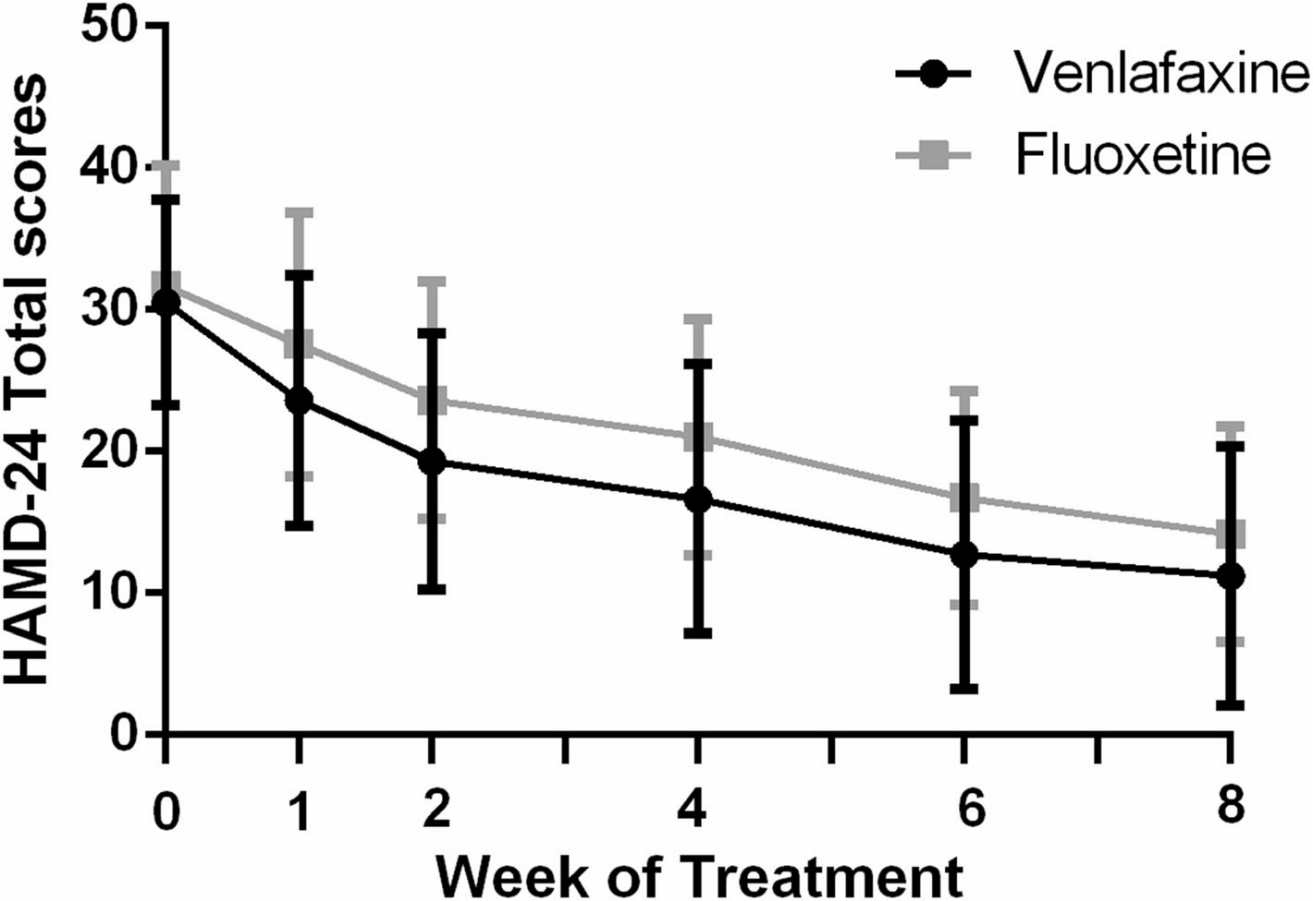
24-item Hamilton Depression Rating Scale (HAMD-24) total scores over the 8-week treatment phase

The MMRM model revealed differences between the two groups on HAMD-24 total scores at 8 weeks (F_(1,144)_ = 12.0, *P* = 0.001) (Table 2 and Fig. 2). The venlafaxine group decreased significantly compared with fluoxetine group in the HAMD-24 total scores (least-squares mean difference (LSMD) [95% CI]: − 2.22 [− 7.08, − 0.41]). The HAMD-24 total scores were reduced post-treatment for each group (F_(4,137)_ = 68.8, *P* < 0.01). The analyses did not reveal any significant effects for time × treatment interaction on HAMD-24 (F_(4,137)_ = 0.17, *P* = 0.95).

**Table 2:** The mixed effect model analysis results of the two groups of HAMD-24,anxiety/somatization factor score

	Group	Time	Research center	Research center*Group	Time*group
HAMD(8w, FAS)					
F	12.0	68.8	3.04	0.01	0.17
P	0.001	< 0.01	0.08	0.92	0.95
HAMD(8w, PP)					
F	8.7	64.1	3.2	0.05	0.03
P	0.004	< 0.01	0.08	0.82	0.98
Anxiety/somatization factor(8w, FAS)					
F	14.5	58.4	0.67	0.97	0.9
P	< 0.01	< 0.01	0.42	0.33	0.47

Data were analyzed using a mixed-effect model for repeated measures (MMRM). *HAMD-24* 24-item Hamilton Depression Rating Scale, *FAS* full analysis set, *PP* per-protocol analysis, * interaction

#### Pp

The MMRM model revealed differences between the two groups on HAMD-24 total scores (F_(1,124)_ = 8.7, *P* = 0.004). The venlafaxine group decreased significantly compared with fluoxetine group in the HAMD-24 total scores (least-squares mean difference (LSMD) [95% CI]: − 1.8 [− 6.6, − 0.31]). The HAMD-24 total scores were reduced post-treatment in each group (F_(4,124)_ = 64.1, *P* < 0.01) (Table 2).

### Secondary endpoints

**Fig. 3:**
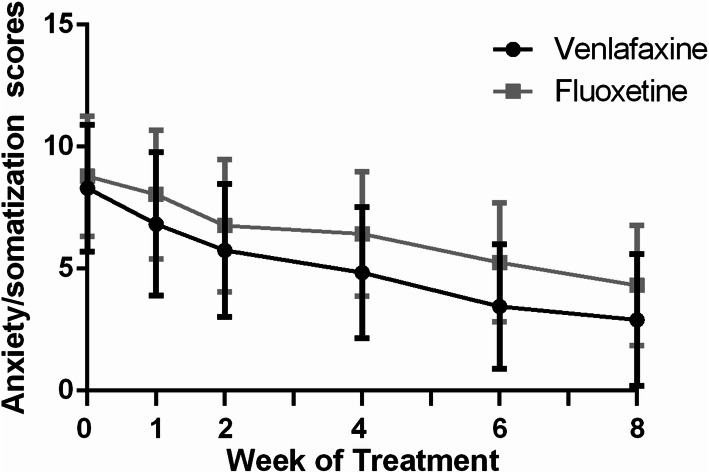
Anxiety/somatization scores over the 8-week treatment phase

The MMRM model revealed differences between the two groups on the total anxiety/somatization factor scores (F_(1,137)_ = 14.5, P < 0.01) (Table 2 and Fig. 3). Venlafaxine group decreased significantly compared with fluoxetine group in anxiety/somatization factor scores (LSMD [95% CI]: − 2.33 [− 2.25, − 0.19]). The anxiety/somatization factor scores were reduced over the course of treatment for each group (F_(4,133)_ = 58.4, P < 0.01).

**Fig. 4:**
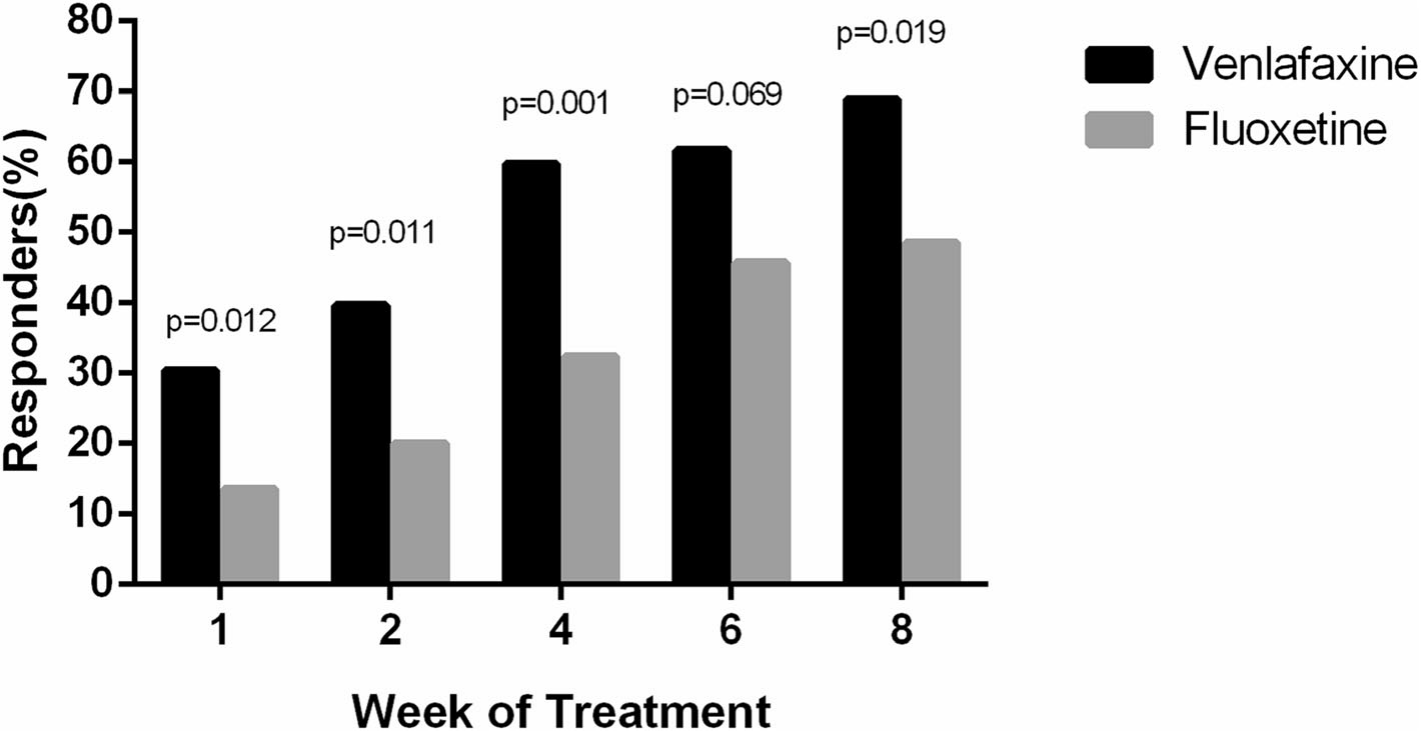
Proportion of patients with “very much or much improved” (CGI-Improvement = 1 or 2) at each follow-up visit

At 8 weeks, the proportion of responders on CGI-I was 68.9% in the venlafaxine group and 48.5% in the fluoxetine group (*P* = 0.019) (Fig. 4).

### Safety and tolerability

Safety analyses were performed in 89 subjects on venlafaxine and 93 on fluoxetine. Twenty-four subjects in the venlafaxine group (27.0%; 31 events) and 30 in the fluoxetine group (32.3%; 44 events) reported treatment-emergent adverse events (TEAEs) (*P* = 0.435). Study drug-related TEAEs were reported by 16 subjects in the venlafaxine group (18.0%, 20 events) and 25 in the fluoxetine group (26.9%, 36 events)(*P* = 0.151). Three subjects in the venlafaxine group (3.4%) and eight in the fluoxetine group (8.6%) discontinued treatment because of TEAEs (*P* = 0.139). All TEAEs were mild or moderate in severity.

The most common drug-related TEAEs reported by the patients in the venlafaxine group were nausea (15.7%), somnolence (6.7%), and dizziness (5.6%). The most common TEAEs in the fluoxetine group were nausea (11.8%), dry mouth (7.5%), dizziness (6.5%), and headache (5.4%). The venlafaxine group exhibited a significantly higher incidence of leukopenia, constipation, and difficulty urinating. The fluoxetine group had a higher incidence of increased intraocular pressure (Table 3). No SAE was reported. One participant reported a patellar fracture, and one participant in the fluoxetine group reported cervical spondylosis (not study drug-related).

**Table 3:** Adverse events

Event, n (%)	Venlafaxine (*n* = 89)	Fluoxetine (*n* = 93)	*P*
Nausea	14 (15.7)	11 (11.8)	0.445
Somnolence	6 (6.7)	3 (3.2)	0.274
Dizziness	5 (5.6)	6 (6.5)	0.813
Dry mouth	4 (4.5)	7 (7.5)	0.391
Headache	4 (4.5)	5 (5.4)	0.784
Hand shake	3 (3.4)	2 (2.2)	0.615
Sweating	2 (2.2)	1 (1.1)	0.535
Constipation	2 (2.2)	0	–
Diarrhea	1 (1.1)	3 (3.2)	0.334
Difficulty urinating	1 (1.1)	0	–
Leukopenia	1 (1.1)	0	–
Increased intraocular pressure	0	1 (1.1)	–

## Discussion

### Principal findings

Previous studies comparing SNRIs and SSRIs in the treatment of postmenopausal MDD have several limitations [8–11, 19, 20]. Therefore, the aim of this study was to compare the efficacy and safety of venlafaxine vs. fluoxetine in the treatment of postmenopausal MDD. The results suggested that venlafaxine was well tolerated, leading to greater improvement than fluoxetine in the treatment of postmenopausal MDD. The safety profile was comparable between the two groups.

### Results

This is the first multicenter, randomized, controlled trial that evaluated the efficacy of fluoxetine vs. venlafaxine in women with postmenopausal MDD. The symptoms of depression significantly improved in both groups; however, venlafaxine was superior to fluoxetine in controlling depression symptoms. Our results showed that menopause affected SSRI’s antidepressant effect, which is consistent with other studies comparing the effect of SNRIs and SSRIs in menopausal depression [11]. SNRIs may have a consistent antidepressant effect in women across different ages and menopausal staging. In their study on sleep-related issues, Davari-Tanha et al suggested that venlafaxine is more efficacious than citalopram in the treatment of depression in postmenopausal women [21]. The results from the present study confirm and extend these findings. On the other hand, Soares et al found no significant differences in the efficacy of SNRIs and SSRIs in their study on the treatment of postmenopausal women with MDD [22]. The main reason for these inconsistencies might be the high doses of desvenlafaxine (100–200 mg/d) that did not necessarily confer a greater magnitude of efficacy but were associated with greater TEAEs. By contrast, the doses in our study, which were prescribed according to the physicians’ decision, all fell within the recommended doses, i.e., 75–225 mg/d for venlafaxine and 20–60 mg/d for fluoxetine.

There are other possible explanations for the differences in drug efficiency. First, the pharmacological profiles of the drugs could explain the clinical differences between them. Venlafaxine is an antidepressant with a mechanism of action that is believed to involve the inhibition of the uptake pumps for 5-HT and NE with inhibition of NE uptake, which is particularly relevant at high doses [23]. Fluoxetine is an antidepressant of the SSRI class [24] that mainly inhibits 5-HT. Second, estrogens decline in postmenopausal women [25], and augments the response to SSRIs in female patients with MDD [26, 27]. Animal studies support the notion that estrogens increase serotonergic activity, which could explain why postmenopausal patients have a poor response to SSRIs. Estrogen can increase 5-HT by decreasing the expression of monoamine oxidases-A (MAO-A) activity, the enzymes responsible for the degradation of 5-HT [28, 29]. Estrogens increase the activity of tryptophan hydroxylase, the rate-limiting enzyme involved in the synthesis of 5-HT, resulting in an increase in overall 5-HT availability [30, 31]. Estrogens also decrease the serotonin reuptake transporter; this transporter, located presynaptically, is very important in the elimination pathway of 5-HT from the synaptic cleft [32]. Taken together, these points could explain why menopausal depressed women were less responsive to SSRIs.

In the present study, the venlafaxine-treated group displayed a statistically significant earlier improvement of MDD symptoms compared with fluoxetine, which is consistent with a previous study [33]. Venlafaxine has an acute onset of down-regulation of β-adrenergic receptors, which might be a mechanism underlying the early onset of action [34]. It will be necessary to determine the exact mechanisms of the early improvement of depressive symptoms in menopausal women when using venlafaxine.

The present study also showed that venlafaxine significantly improved anxiety and somatization compared with fluoxetine. These findings are in line with a previous study in which venlafaxine was superior to fluoxetine in improving anxiety symptoms [33]. Similarly, a pooled analysis from five double-blind, randomized studies showed that venlafaxine is superior to fluoxetine in improving anxious symptoms [35]. The AEs and TEAEs of venlafaxine and fluoxetine were similar. Treatment discontinuation due to AEs occurred at low incidence, thus suggesting that the two drugs were well tolerated. Also, all TEAEs in the present study were mild or moderate in severity, which is again consistent with previous studies [35].

### Clinical implications

The overall health and well-being of middle-aged women have become a major public health concern around the world. According to the current life expectancy, women spend almost a third of their life being menopausal and estrogen-deficient [36]. More than 80% of the women experience physical or psychological symptoms in the years when they approach menopause, with various distress and disturbances in their lives, leading to a decreased quality of life [37]. Women are about twice as likely as men to develop depression during their lifetime, and the postmenopausal period is associated with a higher vulnerability to depression among female patients [38–40]. Postmenopausal women are more likely to have suicidal ideation and poorer physical functioning than premenopausal and perimenopausal women [41]. Hence, it is essential to optimize pharmacologic options for the treatment of patients with postmenopausal MDD. This study suggests that women with postmenopausal major depressive disorder might benefit more from an SNRI than from an SSRI, with a more rapid and better response to treatment. The results provide some clues to optimize antidepressant pharmacotherapy for postmenopausal MDD.

### Strengths and limitations

This was a prospective trial, and it was finally adequately powered to verify the hypothesis; however, there are still some limitations. First, we did not measure estrogen levels at baseline. Future studies should measure estrogens, which could provide a new perspective towards understanding estrogen’s influence on antidepressants. Second, 8 weeks of antidepressant treatment were insufficient to evaluate the long-term effects, and a longer follow-up period is required. Third, we only compared two active treatments without a placebo. Fourth, Menopause-Related Symptoms were not used to evaluate hot flashes, night sweats, and other menopausal symptoms in this study. Fifth, this study did not include treatment-resistant depression (TRD) in postmenopausal depression; research in this area will be strengthened in the future. Sixth, the drop-out rate was high in this study. Finally, we did not recruit 200 patients as per the sample size calculation This non-significant finding might be due to a type II error, underlining the need for replication. Only 189 were included due to time limit and economic resources.

## Conclusions

This randomized controlled trial evaluated the comparative efficacy of SNRIs vs. SSRIs for the treatment of MDD in postmenopausal patients. After 8 weeks of treatment, the efficacy of venlafaxine is superior to fluoxetine in the treatment of postmenopausal MDD. Venlafaxine and fluoxetine were safe and well-tolerated. Future, large-scale clinical trials are warranted to evaluate the efficacy and tolerability of SNRIs and SSRIs in the treatment of postmenopausal MDD.

## Data Availability

The datasets used and/or analyzed during the current study are available from the corresponding author.

## Abbreviations

AEs: Adverse events
CGI-I: Clinical Global Impressions-Improvement
CI: Confidence interval
DSM-IV: Diagnostic and Statistical Manual of Mental Disorders-IV
ECG: Electrocardiogram
ECT: Electroconvulsive therapy
FAS: Full analysis set
HAMD-24: 24-item Hamilton Depression Rating Scale
LSMD: Least-squares mean difference
PP: Per-protocol analysis
MAO-A: Monoamine oxidases-A
MDD: Major depressive disorder
MINI: Mini International Neuropsychiatric Interview
mITT: Modified intention-to-treat
MMRM: Mixed-effect model for repeated measures
SD: Standard deviation
SNRIs: Serotonin-norepinephrine reuptake inhibitors
SS: Safety set
SSRIs: Selective serotonin reuptake inhibitors
